# Accidental Swallowing of a Hypodermic Needle

**DOI:** 10.5005/jp-journals-10005-1032

**Published:** 2009-08-26

**Authors:** Nikhil Srivastava, IK Pandit, Vineeta Nikhil, Neeraj Gugnani

**Affiliations:** 1 Professor, Department of Pedodontics, Preventive and Community Dentistry, DAV (C) Dental College and Hospital Yamunanagar- 135001, Haryana, India; 2 Principal, Professor and Head, Department of Pedodontics, Preventive and Community Dentistry, DAV (C) Dental College and Hospital, Yamunanagar-135001, Haryana, India; 3 Professor and Head, Department of Conservative Dentistry and Endodontics, DAV (C) Dental College and Hospital Yamunanagar- 135001, Haryana, India; 4 Professor, Department of Pedodontics, Preventive and Community Dentistry, DAV (C) Dental College and Hospital Yamunanagar- 135001, Haryana, India

**Keywords:** Accidental, needle, swallowing.

## Abstract

This unusual case report describes the accidental swallowing of a hypodermic needle by a patient during a conservative procedure, which though safely discharged in the stool after 24 hours but emphasizes the use of two major preventive measures namely, rubber dam and oral packing during all endodontic and conservative procedures to prevent the occurrence of such unfortunate incidence.

## INTRODUCTION

Despite of the best efforts at prevention, small objects like inlays, alloys, burs, metal crowns and endodontic instruments may fall into the oropharynx of the patient with subsequent swallowing or aspiration. The introduction of sit down, four hand dentistry in which the patient is placed in supine or semi-supine position during treatment has increased the possibility of such occurrence.^1^

It has been widely accepted that the use of rubber dam is essential to maintain a sterile and clean operating field during conservative or endodontic therapy and to avoid the ingestion or aspiration of small devices used.^2^ Swallowing or aspiration of files and reamers has been described in dental literature. The instrument that may go through the digestive tract of a patient in a period ranging from a few days to a month^3^,^4^ or the instrument may lie in the stomach,^5^ duodenum,^6^ colon^7^ or appendix^8^ in which case surgery may be necessary to remove it because of infection complications.

The present case reports with the accidental swallowing of a hypodermic needle during a conservative procedure which was discharged in the stool without any complication.

## CASE REPORT

**Fig 1:**
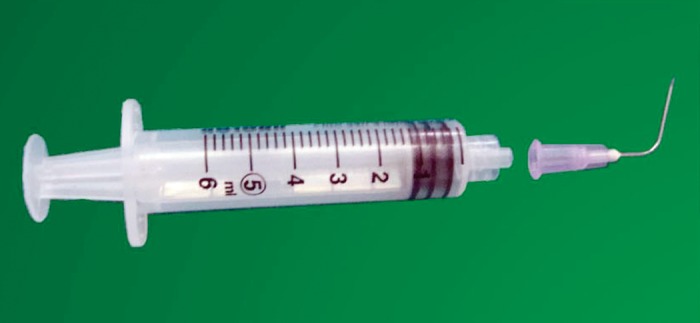
The ‘bend’ hypodermic needle, similar to the needle which was swallowed

An apprehensive 16 years old male child patient was brought to a Dental Emergency of DAV (C) Dental College and Hospital, Yamunanagar by a private practitioner of the local area. The practitioner disclosed that the patient reported to his clinic with fissure caries in the right mandibular first molar. After diagnosis, he prepared the cavity with airotor but for washing of the prepared cavity, a disposable syringe with ‘bend needle’ was used, as the three way syringe of the dental unit was not working (Fig. 1). During washing, the bend needle detached from the syringe and was accidentally swallowed by the patient.

**Fig. 2:**
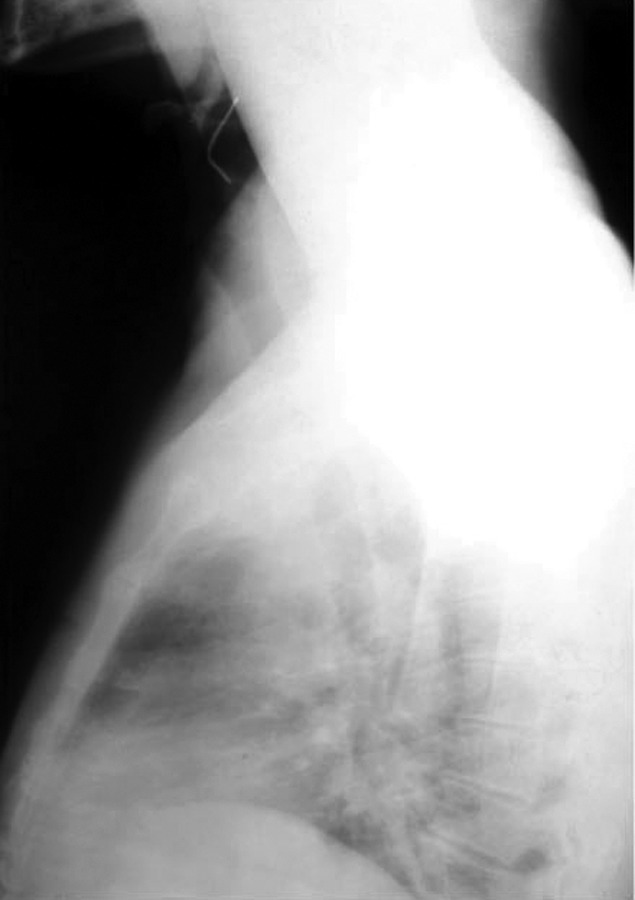
Roentgenogram of cervical region showing the ‘bend’ hypodermic needle lying just below the cricoid cartilage

In the emergency department, the patient was immediately evaluated and no signs of respiratory obstruction (e.g. dyspnea or cyanosis) were observed though some gagging, mild pain in the neck during swallowing and slight increase in respiratory and puise rate were observed. He was examined by the General surgeon who advised Xrays of cervical region which showed the needle lying in the throat just above the cricoid cartilage (Fig. 2).

**Fig. 3:**
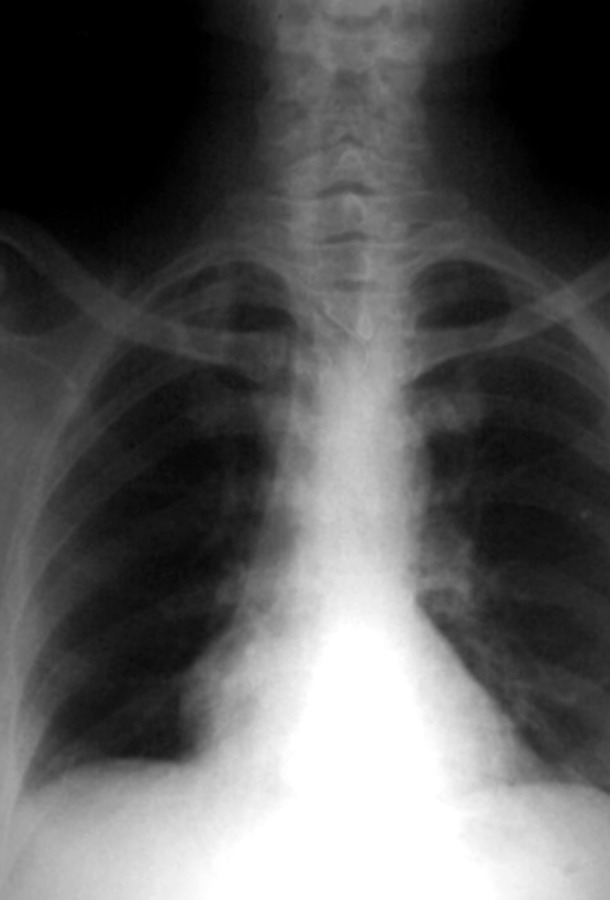
Posteroanterior chest radiograph showing the swallowed needle

**Fig. 4:**
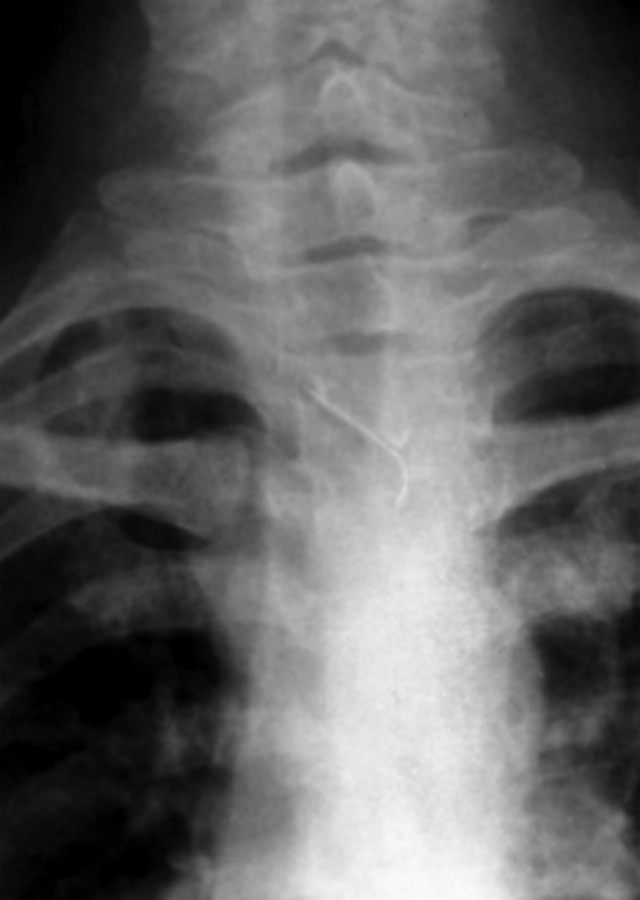
Enlarged view of needle lying in rnidthorax

An ENT surgeon was called who tried to remove the needle with the help of Laryngoscope and Magill forceps under short acting general anesthesia but could not succeed. Another X-ray of the cervical region was taken to exactly locate the position of the needle. It was thought that during manipulation, the needle might have shifted down. To confirm its further position, PA view chest was taken which showed the needle was lying in the mid thorax (Figs 3 and 4).

The patient was kept under observation for more than 6 hours and instructions given to the patient and his parents to observe the stool for needle. No medication prescribed but was advised to take fibrous food. The needle was effectively discharged in the stool 24 hours later with no pain or discomfort during this period.

## DISCUSSION

It has been stated imperatively that the use of rubber dam is mandatory in all endodontic and conservative procedures to implement a sterile operating technique and to avoid the risk of loosing small instruments down the trachea or esophagus.^2^

Barkmeier and Colleagues^8^ stated that two major preventive measures to minimize the occurrence of swallowed foreign objects are the proper use of rubber dam and oral packing. But when the above measures are not employed and an object enters the oropharynx of a patient who is in supine or semi-supine position, do not allow the patient to sit up rather position the chair into a head down (Trendelenburg) position so that the object can come back into the oral cavity due to the gravity and can be taken out. If the patient has swallowed it, a series of radiographs of cervical, chest and abdomen are required to locate its position.

Sharp devices like files, burs, broaches and needles are especially hazardous and can produce serious health problems like abscesses, fistulas, peritonitis or septicemia as these may lodge in duodenum or colon (peritonitis) and in caecum (acute appendicitis).^5^,^8^

It is therefore, advised to use rubber dam always before any conservative or endodontic procedures or at least oral packing (such as gauge dressing in throat) to avoid accidental swallowing of foreign objects. It is also advised to always use lock syringes especially when operating in the oral cavity so that detachment of the needle from the syringe during injection can be prevented.
